# Gene Organization in Rice Revealed by Full-Length cDNA Mapping and Gene Expression Analysis through Microarray

**DOI:** 10.1371/journal.pone.0001235

**Published:** 2007-11-28

**Authors:** Kouji Satoh, Koji Doi, Toshifumi Nagata, Naoki Kishimoto, Kohji Suzuki, Yasuhiro Otomo, Jun Kawai, Mari Nakamura, Tomoko Hirozane-Kishikawa, Saeko Kanagawa, Takahiro Arakawa, Juri Takahashi-Iida, Mitsuyoshi Murata, Noriko Ninomiya, Daisuke Sasaki, Shiro Fukuda, Michihira Tagami, Harumi Yamagata, Kanako Kurita, Kozue Kamiya, Mayu Yamamoto, Ari Kikuta, Takahito Bito, Nahoko Fujitsuka, Kazue Ito, Hiroyuki Kanamori, Il-Ryong Choi, Yoshiaki Nagamura, Takashi Matsumoto, Kazuo Murakami, Ken-ichi Matsubara, Piero Carninci, Yoshihide Hayashizaki, Shoshi Kikuchi

**Affiliations:** 1 Division of Genome and Biodiversity Research, National Institute of Agrobiological Sciences, Tsukuba, Ibaraki, Japan; 2 Hitachi Software Engineering, Shinagawa-ku, Tokyo, Japan; 3 Laboratory of Genome Sequencing and Analysis Group, Foundation for Advancement of International Science (FAIS), Tsukuba, Ibaraki, Japan; 4 Nara Institute of Science and Technology (NAIST), Ikoma, Nara, Japan; 5 Genome Exploration Research Group, Genomic Sciences Center, RIKEN Yokohama Institute, Yokohama, Kanagawa, Japan; 6 Genome Science Laboratory, RIKEN Wako Institute, Wako, Saitama, Japan; 7 Institute of the Society for Techno-innovation of Agriculture, Forestry and Fisheries, Tsukuba, Ibaraki, Japan; 8 Plant Breeding, Genetics and Biotechnology Division, International Rice Research Institute, DAPO, Metro Manila, Philippines; Umeå Plant Science Centre, Sweden

## Abstract

Rice (*Oryza sativa* L.) is a model organism for the functional genomics of monocotyledonous plants since the genome size is considerably smaller than those of other monocotyledonous plants. Although highly accurate genome sequences of *indica* and *japonica* rice are available, additional resources such as full-length complementary DNA (FL-cDNA) sequences are also indispensable for comprehensive analyses of gene structure and function. We cross-referenced 28.5K individual loci in the rice genome defined by mapping of 578K FL-cDNA clones with the 56K loci predicted in the TIGR genome assembly. Based on the annotation status and the presence of corresponding cDNA clones, genes were classified into 23K annotated expressed (AE) genes, 33K annotated non-expressed (ANE) genes, and 5.5K non-annotated expressed (NAE) genes. We developed a 60mer oligo-array for analysis of gene expression from each locus. Analysis of gene structures and expression levels revealed that the general features of gene structure and expression of NAE and ANE genes were considerably different from those of AE genes. The results also suggested that the cloning efficiency of rice FL-cDNA is associated with the transcription activity of the corresponding genetic locus, although other factors may also have an effect. Comparison of the coverage of FL-cDNA among gene families suggested that FL-cDNA from genes encoding rice- or eukaryote-specific domains, and those involved in regulatory functions were difficult to produce in bacterial cells. Collectively, these results indicate that rice genes can be divided into distinct groups based on transcription activity and gene structure, and that the coverage bias of FL-cDNA clones exists due to the incompatibility of certain eukaryotic genes in bacteria.

## Introduction

Rice (*Oryza sativa* L.) is a model organism for functional genomics of monocot plants because the genome size is smaller than those of other monocot plants. Highly accurate genome sequences of rice are available [1]–[Bibr pone.0001235-Yu1] [Bibr pone.0001235-Yu2] [4]. Genome sequences of rice (*O. sativa* ssp. *japonica* cv. Nipponbare) have been assembled independently by The Institute for Genome Research (TIGR), International Rice Genome Sequencing Project (IRGSP), and Beijing Genomics Institute (BGI). In addition, full-length complementary DNA (FL-cDNA) sequences [5] and expressed sequence tags (ESTs) [6]–[Bibr pone.0001235-Zhou1] [8] from rice have served as valuable resources for genomic and genetic studies.

The rice genome sequences assembled by TIGR (TIGR Pseudomolecules) have been revised to Pseudomolecules Release 4 (TIGR4) [9]. TIGR4 was subsequently used to improve the accuracy of predicted gene loci, model genes and gene annotation. (see TIGR OSA1: http://www.tigr.org/tdb/e2k1/osa1/index.shtml) [9]. In TIGR OSA1, the transcriptome information compiled from numerous data of FL-cDNA, ESTs, Massively Parallel Signature Sequencing (MPSS), and Serial Analysis of Gene Expression (SAGE) were integrated with proteome information. This comprehensive information eventually led to the confirmation of gene expression at 24 799 loci. The genes in TIGR OSA1 were annotated by semi-automatic and manual methods. The OSA1 information has been used for gene classification and assimilation into physical map data [10], [11] and has accelerated genomic analysis of rice [12], [13].

Builds 3 and 4 of IRGSP Pseudomolecules (IRGSP3 and IRGSP4) are other versions of the rice genome sequences [4] available at the Rice Annotation Project Database (RAP-DB: http://rapdb.lab.nig.ac.jp/) [14], [15]. Gene loci in IRGSP3 and IRGSP4 were determined using only cDNA sequences (FL-cDNA and ESTs, combined ESTs) derived from rice and other cereals. Gene loci in RAP-DB were verified by cDNA analysis, but other predicted loci lacking cDNA support were excluded from the database. The genes in IRGSP3 and IRGSP4 were annotated manually.

Both TIGR Pseudomolecules and IRGSP Pseudomolecules assembled from the same source of bacterial and P1 artificial chromosome clones were constructed by map-based methods. The rice genome sequences for cultivars 93-11 and Nipponbare assembled by BGI were determined by the whole-genome shotgun method (available at RISe: http://rise.genomics.org.cn/) [16]. The genome of 93-11 (*O. sativa* ssp. *indica*) was sequenced by BGI [2], whereas that of Nipponbare was done by Syngenta [1]. The RISe database provides predicted gene loci, expression data from genome tiling arrays, and the mapped positions of FL-cDNA and ESTs [16], [17].

FL-cDNA collections have been established for several organisms [18]–[20]. The cDNA information was extensively used to determine gene annotation, structures, and the start and the end sites of transcription. The cDNA information also is indispensable for validation of gene function by reverse genetics. The FL-cDNA collection for rice also has been used to complement the information obtained by genome sequencing. The first report of a rice FL-cDNA collection published in 2003 [5] described the characteristics and annotation of 28K FL-cDNA sequences. The collection has eventually expanded to 578K FL-cDNA clones, among which 35K cDNA clones were completely sequenced and annotated (available at Knowledge-based Oryza Molecular biological Encyclopedia (KOME): http://cdna01.dna.affrc.go.jp/cDNA/).

The informative data from MPSS [21], SAGE [22], [23], and genome tiling arrays [17] can serve as additional resources complementary to the rice genome sequences, although the individual approaches may not be able to provide information entirely sufficient for gene prediction. MPSS and SAGE provide supportive evidence for gene expression. The two approaches may identify the start or end of transcription sites, but cannot reveal gene structures. Genome tilling arrays can define exon-intron structures [17], [24], but not the start and end of individual transcripts or alternative transcripts. In contrast, as already described, the large collection of annotated FL-cDNA sequences of rice available from several databases has been used as a comprehensive resource for various analyses of gene structure and function.

The main objective of this report is to more completely describe the features of the FL-cDNA sequences mapped in the rice genome. We examined global characteristics such as the coverage of full-length cDNA clones among predicted rice genes, and the structural and expression characteristics of genes which were not identified by the gene prediction programs. The results suggest that the cloning efficiency of the FL-cDNA was greatly influenced by transcriptional activity and the structure of the encoded gene. In addition, the results also revealed that the levels of transcription activity are significantly associated with the position within the genome and the structure of individual genes, and that protein domains encoded in individual genes may act as a significant factor causing bias in cloning of FL-cDNA.

## Results and Discussion

### 1. Results of mapping cDNA to five currently available genome assemblies

Five hundred seventy eight thousand rice (ssp. *japonica*, cv. Nipponbare) FL-cDNA clones (DDBJ accession number: completely sequenced FL-cDNA : AK058203–068528, AK068530–068912, AK068914–70720, AK070722–074028, AK098843–112119, AK119160–122186, AK240633–243692, One-pass sequences of FL-cDNA, 5′ end: CI285358-311811 and CI563340–778739, 3′end: CI000001-285357 and CI311812–563339) were mapped to five genome assemblies: TIGR4, IRGSP3, IRGSP4, the Nipponbare genome determined by Syngenta, and the 93-11 genome by BGI (Table 1, S1). The mapping criteria were >95% identity and >90% coverage. For proper comparison between the assemblies, the results of mapped cDNA for the respective assemblies were compared with those for TIGR4. The numbers of mapped FL-cDNA clones differed among assemblies, with the highest in TIGR4. For some cDNA clones, the orientation in which the clone was mapped onto a chromosome and the chromosome on which the clones were mapped were not consistent among the assemblies.

**Table 1:** FL-cDNA clones mapped to five rice genome assemblies.

	origin sequencing All	*japonica* genome				*indica* genome
		Map-base cloning			whole shotgun	
		TIGR	IRGSP4	IRGSP3	Syngenta	93-11
**FL-cDNA**	35,187	32,775	32,745	32,640	31,928	30,354
**5endFLEST**	241,854	212,598	212,539	211,564	208,606	199,001
**3endFLEST**	536,885	483,657	484,358	482,909	482,665	465,775
**FL-cDNA locus**	Chr1	4,026	4,021	4,039	4,050	3,940
	Chr2	3,196	3,198	3,215	3,186	3,153
	Chr3	3,569	3,567	3,566	3,597	3,607
	Chr4	2,531	2,530	2,534	2,477	2,493
	Chr5	2,313	2,305	2,310	2,338	2,329
	Chr6	2,292	2,293	2,290	2,262	2,266
	Chr7	2,183	2,185	2,193	2,165	2,021
	Chr8	1,933	1,934	1,939	1,912	1,827
	Chr9	1,605	1,605	1,574	1,545	1,515
	Chr10	1,538	1,528	1,536	1,502	1,416
	Chr11	1,685	1,683	1,675	1,486	1,333
	Chr12	1,693	1,692	1,705	1,523	1,435
	Chr0 ^(a)^				434	497
	Total	28,564	28,541	28,576	28,477	27,832
**Comparison of FL-cDNA mapping with TIGR4**		Both mapped	32730	32623	31741	30162
		Same Chr-Same Strand	32646	32611	30422	28760
		Same Chr-Reverse Strand	80	10	317	335
		Differential Chr.	4	2	1002	1067
		Mapped on only TIGR	45	152	1034	2613
		Unmapped on only TIGR	15	17	187	192
		Both unmapped	2397	2395	2225	2220
		**mapped on all assemblies**			29925	
		**unmapped on all assemblies**			2186	

* : sequence-assembled contigs that were not localized to one of the 12 chromosomes.

Of the 32 775 completely sequenced FL-cDNA clones mapped in TIGR4, 29 925 were also mapped in all of the other assemblies, however, the number of clones commonly mapped in both TIGR4 and a given assembly differed (Table 1). The maximum and minimum numbers of common clones were 32 730 in IRGSP4 and 30 162 in 93-11, respectively. The number of mapped clones was greater in the *japonica* rice genomes than in the *indica* genome which might reflect differences in the genome sequences between subspecies. The number of common clones between TIGR4 and IRGSP4 was close to that between TIGR4 and IRGSP3, and both numbers were greater than the number of common clones between TIGR4 and the Syngenta sequence. This suggests that the differences in numbers of common clones may have resulted from differences in sequencing methods adopted in the assemblies (TIGR4 and IRGSP by the map-based method; Syngenta sequence by the whole-genome shotgun method).

Mapping of 578K FL-cDNA clones identified about 28 500 loci in the *japonica* genome and 27 800 loci in the *indica* genome. A total of 29 925 completely sequenced FL-cDNAs were mapped in any genome assemblies and more than 90% of the FL-cDNAs were mapped in all five assemblies (Table 1). So, we decided to use only the mapping results of TIGR4 for further analyses and to not use results from the other assemblies. The number of predicted loci was about 56K which was sufficient for our data analysis, but probably not sufficient to reach complete accuracy of gene prediction and annotation of TIGR4.

### 2. Classification of loci according to FL-cDNA mapping

A total of 55 890 gene loci were predicted in the rice genome according to TIGR OSA1 release 4. Mapping of FL-cDNA clones on TIGR4 revealed that 533 667 FL-cDNA clones were derived from 28 564 FL-cDNA loci (Table 2). FL-cDNA loci were cross-referenced with TIGR4 loci to examine the overlaps between the two locus groups. According to the sources of mapped loci and the occurrence of overlap, the loci were classified as follows: 1) when a FL-cDNA locus overlapped with a TIGR4 locus, the FL-cDNA locus was defined as FL-AE (annotated expressed), and the TIGR4 locus was defined as coding-sequence-AE (CDS-AE); 2) a FL-cDNA locus that did not overlap with any TIGR4 locus was defined as FL-NAE (non-annotated expressed); and 3) a TIGR4 locus that did not overlap with any FL-cDNA locus was defined as CDS-ANE (annotated non-expressed). Based on the definitions, the loci were classified into 23 117 FL-AE, 23 193 CDS-AE, 5 447 FL-NAE, and 32 697 CDS-ANE (Tables 2, S2a).

**Table 2:** Comparisons of FL-cDNA loci and TIGR4 loci

		Class		
		AE	NAE	ANE
**TIGR CDS**		23193	0	32697
**FL-locus**		23117	5447	0
**mapping information**	**FL-cDNA**	29808	2967	0
	**5endFLEST**	201343	11255	0
	**3endFLEST**	465816	17481	0
	**FL-clones**	511817	21850	0

### 3. Characterization of loci based on FL-cDNA mapping

The classification of loci as defined above raised questions concerning whether any characteristic distinctions exist between FL-AE and FL-NAE, and why FL-NAE loci were not predicted in TIGR4. To answer these questions, we analyzed the structures of genes belonging to the respective groups.

### Open reading frame of FL-cDNA clones mapped on genome

**Figure 1:**
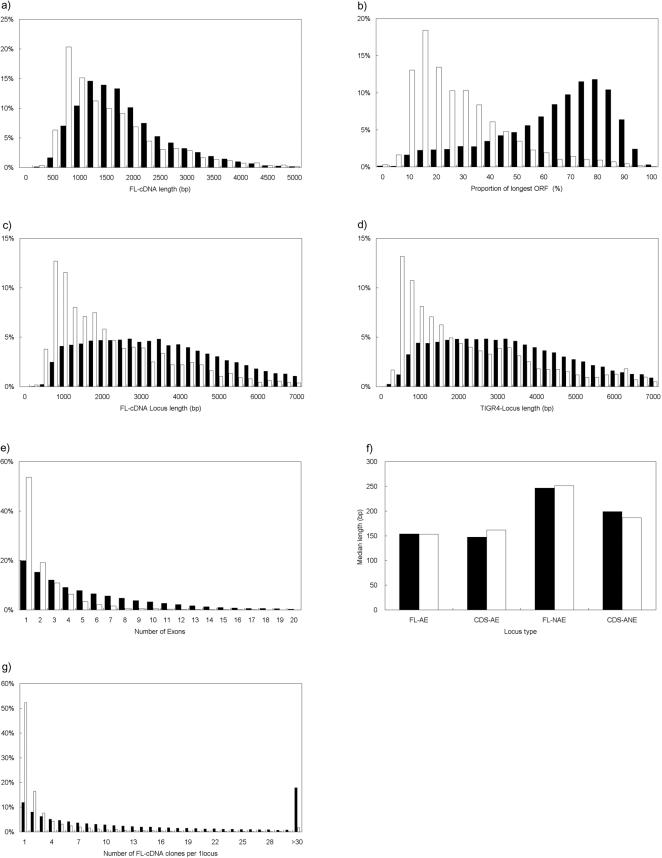
Gene structure analysis in rice. (a) The length distribution of FL-cDNA for FL-AE (black) and FL-NAE (white). (b) The distribution of open reading frame (ORF) proportions for FL-AE (black) and FL-NAE (white). (c) The distribution of FL-cDNA locus lengths for FL-AE (black) and FL-NAE (white). (d) The distribution of locus lengths for CDS-AE (black) and CDS-ANE (white) in TIGR4. (e) The distribution of the number of exons for FL-AE (black) and FL-NAE (white). (f) The distribution of exon (black) and intron (white) lengths for the respective locus types. (g) The distribution of the number of FL-cDNA clones mapped per single FL-AE (black) and FL-NAE (white) locus.

We mapped 32 775 FL-cDNA at 22 943 FL-cDNA loci (FL-AE, 20 324; FL-NAE, 2619) (Table S2b). The numbers of FL-cDNA mapped to FL-AE and FL-NAE were 29 808 and 2967, respectively (Table 2). The median lengths of the FL-cDNA mapped to FL-AE and FL-NAE were 1540 and 1173 bp, respectively (Figure 1a, Table 3).

**Table 3:** Structural characteristics of locus types

	FL-cDNA length (median)	ORF ratio (median)	Locus length (median) (a)	Variation of locus length	Number of exons (average) (b)	Exon length (median) (b)	Intron length (median) (c)	Ave.nunber of mapped FL-cDNA clones
**FL-AE**	1540	66%	3354	Rich	5.3	154	153	22.3
**FL-NAE**	1173	21%	1727	Poor (short)	2.4	247	251	4.1
**CDS-AE**	-	-	3173	Rich	5.8	147	162	-
**CDS-ANE**	-	-	1643	middle	3.9	199	186	-

a : For the calculation of locus lengths, we used the maximum lengths of individual loci.

b : Exons shorter than 10 bp were excluded from the analysis. Thus, the definition of an exon in FL-cDNA loci differs from that in TIGR OSA1.

c : Introns shorter than 10 bp were excluded from the analysis. Thus, the definition of an intron in FL-cDNA loci differs from that in TIGR OSA1.

The proportions of the length of the longest open reading frames (ORF) to that of FL-cDNA were also considerably different between FL-cDNA mapped to FL-AE and FL-NAE. The median proportion of the longest ORF in FL-AE was 66%, versus only 21% in FL-NAE (Figure 1b, Table 3). The results indicate that FL-NAE clones generally encode shorter peptides, and that the ORF lengths differ considerably between FL-AE and FL-NAE (based on the clone length×ORF proportion).

### Locus length

The start and end sites for transcription were determined in 24 164 loci (FL-AE, 21 263; FL-NAE, 2901) out of 28 564 FL-cDNA loci. The distance between the start and end sites (i.e., locus length) was calculated based on TIGR4. The median locus lengths were considerably different between FL-AE and FL-NAE, with lengths of 3354 and 1727 bp, respectively (Figure 1c, Table 3). The average proportion of locus length to FL-cDNA clone length was greater than 2 for FL-AE, but less than 1.5 for FL-NAE.

The median locus lengths for CDS-AE and CDS-ANE were 3173 and 1643 bp, respectively (Figure 1d). The locus lengths of CDS-ANE appeared to be more variable than those of CDS-AE. The patterns of locus length variation for CDS-AE and CDS-ANE were similar to those for FL-AE and FL-NAE, respectively (Figure 1c,d, Table 3). We could not compare the length of FL-cDNA loci with the TIGR4 loci because FL-cDNAs were constructed from coding and 5′ and 3′ end untranslated regions, whereas many gene structures in TIGR4 were predicted only with coding regions. However, based on the results above, we expect that FL-AE and FL-NAE would differ from each other in this characteristic, as would CDS-AE and CDS-ANE.

### Exon-intron structure

The average numbers of exons per FL-AE and FL-NAE were 5.3 and 2.3, respectively (Figure 1e, Table 3). The frequency of loci with a single exon was highest in both FL-AE and FL-NAE when the loci were distributed according to the numbers of exons. However, the proportions of loci with a single exon were significantly different between FL-AE and FL-NAE, with more than 50% in FL-NAE, and about 20% in FL-AE (Figure 1e). When FL-cDNA loci with one exon were excluded, the ratios between the lengths of exons and introns in individual loci were approximately 1 irrespective of the locus type, but exon and intron lengths were significantly different (P<0.01, calculated by student's t-test) between FL-AE and FL-NAE with median lengths of about 150 and 250 bp, respectively (Figure 1f, Table 3).

We also analyzed the exon-intron structures of CDS-AE and CDS-ANE based on the information at TIGR OSA1. The number of exons was higher in CDS-AE (5.8) than in CDS-ANE (3.9) (Table 3). The exon and intron lengths also significantly differed (P<0.01, calculated by student's t-test) between CDS-AE and CDS-ANE with median exon lengths of 147 and 199 bp, and median intron lengths of 162 and 186 bp, respectively (Figure 1f, Table 3).

### Number of mapped FL-cDNA clones at a locus

FL-NAE accounted for about 19% of the entire FL-cDNA loci, while the proportion of FL-cDNA clones mapped as FL-NAE was only 5% (21 850) of all mapped FL-cDNA clones (533 667) (Table 2). The average numbers of FL-cDNA clones mapped per locus (collection efficiency) were significantly different between FL-AE (22.3 clones) and FL-NAE (4.1) (Figure 1g, Table 3). The collection efficiency was 1 for more than half of the FL-NAE loci (Figure 1g, Table 3), suggesting that FL-cDNA clones derived from FL-NAE are more difficult to collect than those from FL-AE.

### Gene annotation

We analyzed the homology of FL-cDNA mapped on TIGR4 with Arabidopsis CDS in The Arabidopsis Information Resource (TAIR6, http://www.arabidopsis.org/) [25] using the BlastX software. Based on the significance of similarity, FL-cDNA clones were classified into highly-homologous (E-value<10^−50^), low-homologous (10^−50^<E-value<10^−10^) and non-homologous FL-cDNA (E-value>10^−10^). Under these criteria, the numbers of FL-cDNA clones classified as highly-, low-, and non-homologous FL-cDNA were 17 759, 7103, and 7913, respectively. Of these clones, 99.5% of highly-homologous FL-cDNA were mapped to 59% of FL-AE, and 92% of FL-NAE were coding non-homologous genes (Table 4). Thus, the results indicate that nearly all highly-homologous genes were derived from FL-AE, and that most FL-NAE loci encode genes likely to be specific to rice or other monocots. This is consistent with a previous report that some rice genes which do not have homologs in Arabidopsis are similar to genes in the *Sorghum* genome [3].

**Table 4:** The frequency of Arabidopsis homologus gene in each FL-locus

	FL-AE		FL-NAE		Total	
homology (a)	Locus	FLcDNA	Locus	FLcDNA	Locus	FL-cDNA
**HH**	11898	17669	75	90	11973	17759
**LH**	4763	6941	140	162	4903	7103
**NH**	3663	5198	2404	2715	6067	7913
**Total**	20324	29808	2619	2967	22943	32775

a : HH, LH, NH: highly-, low- or non-homologous FL-cDNA with Arabidopsis CDS

### Causes of inconsistency between gene prediction and FL-cDNA mapping

Cross-examination between FL-cDNA and TIGR4 loci revealed the existence of FL-NAE clones. The results from our analyses for the locus structures of FL-AE and FL-NAE suggest possible explanations as to why some expressed genes were not annotated. One possible reason is the characteristic length of ORFs in the different classes of loci. The proportion of the longest ORF in FL-NAE (median ratio of 21%) is significantly lower than in FL-AE (median ratio of 66%) indicating that the transcripts from FL-NAE are more likely to encode either small peptides or no peptide. In the TIGR OSA1, 687 CDS encoding less than 50 amino acids were excluded from the predicted gene model. Thus, even though the number of excluded CDS was less than the number of FL-NAE, FL-cDNA sequences overlapping with the excluded CDS in TIGR4 might have been mapped as FL-AE.

Another possible reason may be the difference in exon-intron structure between FL-NAE and FL-AE. The locus lengths of FL-NAE were generally shorter than those of FL-AE, and more than half of the FL-NAE loci contain only one exon (Figure 1e). Meanwhile, the lengths of both exon and intron in FL-NAE were generally longer than those in FL-AE, CDS-AE and CDS-ANE (Figure 1f). If we consider the structure of FL-AE, CDS-AE and CDS-ANE as the standard type for rice genes, then the structure of many FL-NAE loci may be recognized as an irregular form. Due to the unique features of ORFs and the exon-intron structures in FL-NAE, it may have been difficult to assign proper annotation to the genes in FL-NAE through gene prediction software.

The structural difference between FL-AE and NAE is also similar to that between protein-coding mRNA and mRNA-like non-coding RNA (ncRNA) in mouse [26]. In mouse, the total length of ncRNA is shorter and the exon length is longer than in mRNA. Moreover, more than 70% of ncRNA is constructed from one exon. In our classification, FL-AE overlaps with predicted CDS that encode>50 amino acid sequences, so cDNA mapped on FL-AE originates from protein-encoding mRNA. In addition, the diversity of FL-cDNA lengths between FL-AE and FL-NAE is not large and the proportions of ORF between FL-AE and FL-NAE are reversed (Figure 1a,b, Table 3). So, it seems that the proportion of protein-coding FL-NAE loci is not large and many FL-NAE loci encode mRNA-like ncRNA. Therefore, the structural diversity between FL-AE and FL-NAE may correspond to the difference between protein-coding mRNA and mRNA-like ncRNA in rice. In addition, these results may indicate that the structural difference between protein-coding mRNA and mRNA-like ncRNA are also conserved between plant and mammals.

The gene loci in TIGR4 were categorized into three types by cross-examination between FL-cDNA loci and TIGR4 loci. The collection efficiency varied considerably depending on the locus types (FL-AE = 22.3; FL-NAE = 4.1; CDS-ANE = 0). The difference may be associated with the levels of mRNA or the transcription activity of each locus type. Moreover, the general features of locus structure and the average homology levels with Arabidopsis genes were distinctively different among the locus types. Thus, these findings may indicate an interrelationship among locus structure, transcription activity and the assignability of gene annotation. An association of transcription activity with locus structure has been reported in plants [12]: highly expressed genes have longer primary transcript, ORF, exon and intron sequences and have more exons than low expressed genes. The results from the previous report [12] are consistent with our hypothesis that locus structure affects transcription activity, except that the results for intron length differed from our results. The locus length of FL-AE was greater than that of FL-NAE and the collection efficiency of FL-AE was also greater than that of FL-NAE. Moreover, the cloning efficiency of mRNA-like ncRNA in mouse is lower than for protein-coding mRNA and half of the ncRNA in mouse has an efficiency of 1 [26]. Cloning efficiency features in mouse are also similar to those of FL-NAE which may imply that many FL-NAE loci encode mRNA-like ncRNA. Transcription activity of protein-coding mRNA (FL-AE) is presumed to be higher than that of ncRNA (FL-NAE) and the transcriptional diversity has been confirmed by microarray analysis (see below).

We analyzed the diversity between FL-AE and FL-NAE identified from FL-cDNA mapping and found some differences between FL-AE and FL-NAE. The difference between FL-AE and FL-NAE is similar to that between protein-coding mRNA and mRNA-like ncRNA. In our classification, FL-AE implies a protein-coding locus and FL-NAE is an ncRNA-coding locus which might explain why FL-NAE loci are not predicted in TIGR4. Although gene prediction software can identify loci that encode proteins, it does not detect loci that are transcribed into ncRNA. Therefore, prediction software can not find a FL-NAE locus that encodes ncRNA.

### 4. Validation of gene expression on loci

Transcription from 23K TIGR loci was confirmed by the mapping of FL-cDNA, while another 33K TIGR loci were not confirmed. It seemed that the FL-cDNA libraries did not cover all the rice genes in the genome since the transcription of 3K CDS-ANE was confirmed by other transcriptome resources in TIGR OSA1. To obtain a more accurate evaluation of gene expression from FL-cDNA loci and TIGR4 loci, we designed about 82K 60-mer oligo probes based on TIGR Pseudomolecules Release 2. The TIGR4 loci were remapped according to the revised Pseudomolecules and other additional transcriptome resources such as EST, MPSS and SAGE. The 82K designed probes were remapped in TIGR4 according to our criteria (i.e.>95% identity, >90% coverage, non-redundant highest score). As a result, 63 455 probes were mapped at 43 090 loci (FL-AE, 21 885; FL-NAE, 3540; CDS-ANE, 17 665, see Table S3).

RNA samples were prepared from shoots, roots, panicles, and calluses of Nipponbare (*japonica* variety) and shoots of IR64 (*indica* variety), and the signal intensities were compared among the samples (GEO accession number: GSE7366 and GSE7374). The median and average signal intensities for each locus type were similar among the samples from different tissues of Nipponbare, but the intensity values for the samples from IR64 were generally lower than those from Nipponbare (Table S3). The difference in signal intensities between the samples from Nipponbare and IR64 could be due to the variations in the genome sequence. The apparent signal intensities in the samples from IR64 may have been affected by transcription activity and the variations in the genome sequence; therefore, the data from IR64 were not used in further analyses.

A significant signal (see Materials and Methods) was detected at 93% of the loci identified by the designed probes in at least one of the samples from Nipponbare. The numbers of loci with a significant signal were 21702, 3357, and 14 892 for FL-AE, FL-NAE, and CDS-ANE, respectively (Table S3). The median signal intensity varied among the locus types, with the highest intensity for AE (about 2403), followed by NAE (839) and ANE (237, only about 10% of the AE intensity) (Table S3). The 25% and 75% percentiles of signal intensities for FL-AE were about 800 and 6900, respectively, and the corresponding values for CDS-ANE were 0 and 700, respectively. Moreover, the observed signal profiles showing that the intensity of FL-NAE is lower than that of FL-AE also supports the hypothesis that many FL-NAE code mRNA-like ncRNA [26]

Examination of the median signal intensity and the collection efficiency for each locus type revealed a positive correlation between the signal intensity and the collection efficiency in both FL-AE (R = 0.99) and FL-NAE (R = 0.86) (Table 5). The median signal intensities in FL-NAE were consistently lower than those in FL-AE when the collection efficiencies for the two locus types were within the same range. This positive correlation between signal intensity and cloning efficiency suggests it was difficult to create FL-cDNA clones from CDS-ANE transcripts, because the median signal intensity of CDS-ANE (238) was lower than those of FL-AE and FL-NAE (FL-AE: 852, FL-NAE: 596 in range of collection efficiency 1-2).

**Table 5:** Signal intensities in microarray analysis in relation to the locus types and the collection efficiencies of FL-cDNA.

			Collection efficiency (FL-cDNA clones/locus)			
			1-2	3-5	6-9	10-15
**Signal Intensity**	**FL-AE**	**No. Data ** (a)	16380	14056	12656	12404
		**Average**	3779	4323	5452	6716
		**Median**	852	1421	1984	2572
		**25% percentile**	302	516	761	1010
		**75% percentile**	2568	3758	4723	5985
	**FL-NAE**	**No. Data ** (a)	8768	2680	1400	804
		**Average**	3057	3780	6243	9122
		**Median**	596	1007	1648	2115
		**25% percentile**	196	397	576	609
		**75% percentile**	1826	2729	4397	7059

a : Samples from four tissues of Nipponbare were used for the analysis, thus the numbers of data are the numbers of loci in each category multiplied by four (samples).

The results from microarray analysis indicated that genes identified with the designed probes are expressed at a significant level in at least one of the tissues examined (Table 5), although the possibility of cross-hybridization can not be excluded. To avoid misinterpretation of results due to potential cross hybridization effects, we examined the numbers of loci with significant signal intensity at higher threshold values (significant signal intensity>1000). The number of loci with a significant signal intensity decreased for all locus types as the threshold levels increased. At the threshold, 85% of FL-AE loci (18.6K loci) exhibited a significant signal in at least one of the samples from different tissues, while only 58% (2K loci) of FL-NAE and 27% (4.6K loci) of CDS-ANE exhibited a significant signal. This difference was expected since the median intensities were significantly different among the locus types (Table S3). The difference in the numbers of loci with significant signals among the samples from different tissues was not large (Table 6). The number of loci with a significant signal in respective tissues was also similar to the numbers of loci showing significant signals in all four samples (Table 6). A similar tendency was observed for the number of loci classified as no-significant signal (Table 6). These results suggested that some loci in rice are consistently highly expressed and that some loci are consistently expressed at a low level or perhaps not expressed at all.

**Table 6:** The number of significant signal-detected loci in each locus type

		Shoot	Root	Panicle	Callus	All samples (b)
**FL-AE**	No.locus	21885	21885	21885	21885	21885
	Sig. signal (a)	14438	15560	15894	15602	12021
	No-sig. signal (a)	7447	6325	5991	6283	3234
**FL-NAE**	No.locus	3540	3540	3540	3540	3540
	Sig. signal (a)	1640	1528	1725	1584	1267
	No-sig. signal (a)	1900	2012	1815	1956	1474
**CDS-ANE**	No.locus	17665	17665	17665	17665	17665
	Sig. signal (a)	3862	3316	3453	3187	2589
	No-sig. signal (a)	13803	14349	14212	14478	12983

a : Sig.signal (No-sig. signal) indicates the number of loci with (without) significant signal.

b : The number of loci with significant signals in each of the four samples, or number of loci with no significant signal in each of the four samples.

### Relationship between transcription activity and locus position within the genome

It was previously reported that human housekeeping genes (e.g., tissue-non-specific genes) were mapped to clusters in the genome [27], [28]. In our data (Table 6), most FL-AE appeared as consistently-highly expressed genes, while expression of a large portion of CDS-ANE was consistently low. Therefore, we hypothesized that the respective locus types may exhibit a certain distribution pattern in the rice genome. To test the hypothesis, we examined the frequency of loci belonging to each locus type (FL-AE, FL-NAE, and CDS-ANE) along the genome using a sliding-window analysis (Table S2a). The results of the analysis for the respective locus types and TE-related loci are presented in Figure S1. FL-AE and CDS-ANE were not equally distributed within the genome, and there appears to be a negative correlation between the frequency of FL-AE and CDS-ANE (R = −0.97). The frequency of FL-AE decreased in regions rich in TE-related loci (R = −0.82); whereas, the frequency of CDS-ANE loci increased in these regions (R = 0.79). FL-NAE loci appeared more evenly distributed along the genome, and thus the frequency of FL-NAE did not show any correlations with those of FL-AE and TE-related loci (R = −0.24 and 0.21, respectively). TE-rich regions are generally heterochromatin or centromeric regions [29]. Although transcripts from centromeres and heterochromatin in plants have been reported [3], [29]–[32], these regions appear to be mostly silent with low transcription activity [3]. In addition, centromere and heterochromatic regions of Arabidopsis are highly methylated and pseudogenes which contain homology to coding sequences of transposons also contain many methyleted cytosines. The transcription activity of the gene located on highly methylated region is low [33]. From these findings in Arabidopsis, it can be predicted that transcription activity also is suppressed in the centromere and heterochromatic regions in rice.

We also analyzed the relationship between TE density and transcription activities in FL-AE, FL-NAE and CDS-ANE (Table S4). A negative association between the density of TE-related loci and transcription activity was observed for FL-AE and CDS-ANE, but not for FL-NAE. This result implies transcription activity of many genes may also be influenced by chromatin structure (as TE-density), but FL-NAE may not be affected.

Microarray experiments can directly indicate transcription activity that is not detected by FL-cDNA cloning. Microarray experiments revealed the following: 1) Almost all FL-cDNA clones were derived from transcripts mapped to euchromatic regions; 2) Cloning efficiency was positively associated with transcription activity; 3) Transcription activity was affected by chromatin structure, but the activity of FL-NAE was not affected which implies that regulation of transcription activity may be different between FL-AE and FL-NAE; and 4) Although the signal intensity of 25% CDS-ANE exceeded 1000 in at least one tissue, these genes were not obtained as cDNA clones. That is, cloning efficiency is not only dependent on transcription activity, but also is influenced by other factors.

### 5. Other bias for full-length cDNA cloning

The results presented above suggest the efficiency of FL-cDNA cloning may be dependent on transcription activity to a significant degree. The transcription activity of FL-AE and FL-NAE loci was higher than that of CDS-ANE loci; thus, FL-cDNA from CDS-ANE loci could be difficult to obtain. However, microarray analyses revealed that high signal intensity also was detected from about 15% of CDS-ANE loci (2.6K) in samples from all four tissues (Table 6), suggesting that factors other than transcription activity may affect cloning efficiency. To reveal factors which may be involved, we investigated the cloning efficiency of FL-cDNA from gene groups classified on the basis of their functions.

### Relationship between cloning efficiency and gene function

We examined the coverage of FL-cDNA clones for genes encoding transcription factors (TF) and enzymes for metabolic pathways to see whether there are considerable differences among gene families. The coverage of FL-cDNA clones was determined as the ratio of CDS-AE loci to all CDS loci (hereafter designated as CDS-AE proportion). Based on the Rice Transcription Factor Database version 2.1 (http://ricetfdb.bio.uni-potsdam.de/) [11], 1903 TIGR4 CDS were classified as TF (TF-CDS) representing 65 gene families. Transcription of 1432 TF-CDS loci was supported by detection of the corresponding FL-cDNA clones (CDS-AE). The CDS-AE proportion of the entire TF-CDS was about 75%, but varied among gene families (Table 7). The proportions of TF-CDS for AP2-EREBP (64%), MADS (58%), and GRAS (61%) were significantly lower than the proportion (75%) for the entire TF-CDS (*P*<0.05, calculated by chi-squared test), while those for C3H (86%) and PHD (90%) were significantly higher.

**Table 7:** Coverage of genes encoding transcription factors in the rice FL-cDNA libraries

Family (a)	Total loci	CDS-AE	CDS-ANE	CDS-AE Proportion (b)	Significance (c)
**AP2-EREBP**	164	105	59	64.0	Low
**bHLH**	144	100	44	69.4	
**NAC**	123	84	39	68.3	
**MYB**	121	87	34	71.9	
**C2H2**	102	73	29	71.6	
**WRKY**	97	66	31	68.0	
**HB**	91	72	19	79.1	
**bZIP**	85	70	15	82.4	
**MYB-related**	81	58	23	71.6	
**C3H**	66	57	9	86.4	High
**MADS**	64	37	27	57.8	Low
**GRAS**	54	33	21	61.1	Low
**ABI3VP1**	52	35	17	67.3	
**PHD**	49	44	5	89.8	High
**G2-like**	46	36	10	78.3	
**whole TF-coded CDS**	1903	1432	471	75.2	

a : The classification was based on the Rice Transcription Factor Database version 2.1 (http://ricetfdb.bio.uni-potsdam.de/v2.1/)

b : The ratio was calculated as the number of CDS-AE loci/total number of loci.

c : Significances of difference were examined between the CDS-AE proportion for each gene family and all CDS encoding TF by the chi-squared test. High (Low) represents the cloning efficiency of a TF family was significantly higher (lower) than the collection efficiency for the entire TF.

Based on RiceCyc version 1.1 at the GRAMENE Web site (http://www.gramene.org/pathway/) [10], 2750 TIGR4 CDS were annotated as genes encoding enzymes involved in metabolic pathways. The CDS-AE proportion of the entire CDS involved in metabolic pathways was about 75%, but the proportions varied depending on the pathways (Table 8). The CDS-AE proportion for “removal of superoxide radicals” (DTOX1-PWY, 16.5%) was particularly lower than the proportion for the entire CDS (75%). These results imply that the efficiency of FL-cDNA cloning could be biased depending on the nature of gene functions.

**Table 8:** Coverage of genes associated with metabolic pathways in rice FL-cDNAs libraries

Pathway id (a)	Pathway name (a)	All-CDS	CDS-AE	CDS-ANE	CDS-AE proportion (c)	Significance (d)
**PWY-2881,2901, 2902 ** (b)	cytokinins -glucoside biosynthesis	235	144	91	61.3	Low
**LIPAS-PWY**	triacylglycerol degradation	177	127	50	71.8	Low
**PWY-1081**	homogalacturonan degradation	134	95	39	70.9	Low
**DETOX1-PWY**	removal of superoxide radicals	79	13	66	16.5	Low
**GLUCONEO-PWY**	gluconeogenesis	67	61	6	91.0	High
**PWY-3781**	aerobic respiration	66	39	27	59.1	Low
**TRNA-CHARGING-PWY**	tRNA charging pathway	64	61	3	95.3	High
**P61-PWY**	UDP-glucose conversion	63	60	3	95.2	High
**GALACTMETAB-PWY**	galactose degradation I	54	53	1	98.1	High
**COLANSYN-PWY**	colanic acid building blocks biosynthesis	51	50	1	98.0	High
**PWY-1121**	suberin biosynthesis	51	34	17	66.7	Low
**PWY-3821**	galactose degradation III	51	51	0	100.0	High
**PWY1F-FLAVSYN**	flavonoid biosynthesis	50	24	26	48.0	Low
**CHLOROPHYLL-SYN**	chlorophyll biosynthesis	48	46	2	95.8	High
**P1-PWY**	salvage pathways of purine and pyrimidine nucleotides	44	42	2	95.5	High
**PWY-1861**	formaldehyde assimilation II (RuMP Cycle)	42	40	2	95.2	High
**PWY-2181**	phenylpropanoid biosynthesis	40	23	17	57.5	Low
**Metabolic pathway-related CDS**		2750	2125	625	77.3	

a : The classification was based on the RiceCyc database at the GRAMENE Web site (http://www.gramene.org/pathway/).

b : PWY-2881, PWY-2901, and PWY-2902 share the same genes in the respective pathways.

c : The ratio was calculated as the number of CDS-AE loci/total number of loci.

d : Significances of difference were examined between the CDS-AE proportion in genes in the respective pathway and that of all genes associated with metabolic pathways by the chi-squared test. High (Low) represents that the cloning efficiency of genes for a metabolic pathway was higher (lower) than the collection efficiency for all genes associated with metabolic pathways.

### The structures of protein encoded in cDNA influence cloning efficiency

For broader assessment of the biased cloning of FL-cDNA by gene functions, the CDS-AE proportions were examined for gene families which contain domains categorized at Pfam [34] and InterPro [35] in TIGR OSA1 [9]. A total of 24 247 TIGR4 loci, excluding TE genes, were found containing domains listed in Pfam. About 72% (17 459) of such TIGR4 loci were CDS-AE. The CDS-AE proportions varied depending on the domain types. For example, the proportions of CDS-AE for gene families encoding metabolic enzyme related domains (PF00657, PF00561) are generally higher than those for all loci; whereas, the proportions for some gene families encoding domains for regulatory roles such as protein kinases (PF00069) and receptors (PF00560, PF00931) were lower (Table 9, Table S5a). These results corroborate the observation that cloning efficiencies of FL-cDNA vary depending on the nature of gene functions.

**Table 9:** Coverage of genes with Pfam domains in the rice FL-cDNA libraries

Pfam ID (a)	InterPro ID (a)	Short name	All loci	CDS-AE	CDS-ANE	CDS-AE proportion (b)	Significance (c)	Ecoli (d)
**PF00076**	IPR000504	RNP1_RNA_bd	233	204	29	87.6	High	
**PF00400**	IPR001680	WD40	212	198	14	93.4	High	
**PF00036**	IPR002048	EF_hand_Ca_bd	164	137	27	83.5	High	
**PF00271**	IPR001650	Helicase_C	131	117	14	89.3	High	1
**PF00657**	IPR001087	Lipase_GDSL	113	92	21	81.4	High	1
**PF00515**	IPR001440	TPR_1	108	99	9	91.7	High	1
**PF00226**	IPR001623	DnaJ_N	108	92	16	85.2	High	1
**PF00004**	IPR003959	AAA_ATPase_core	106	94	12	88.7	High	1
**PF00106**	IPR002198	SDR	97	80	17	82.5	High	1
**PF00561**	IPR000073	AB_hydrolase_1	91	81	10	89.0	High	1
**PF00069**	IPR000719	Prot_kinase	1289	892	397	69.2	Low	1
**PF00560**	IPR001611	LRR	890	533	357	59.9	Low	1
**PF00646**	IPR001810	F-box	716	413	303	57.7	Low	
**PF00931**	IPR002182	NB-ARC	427	212	215	49.6	Low	
**PF07197**	IPR010811	DUF1409	253	6	247	2.4	Low	
**PF06654**	IPR009546	DUF1165	231	4	227	1.7	Low	
**PF00098**	IPR001878	Znf_CCHC	226	46	180	20.4	Low	
**PF03578**	IPR005213	HGWP	215	3	212	1.4	Low	
**PF05699**	IPR008906	HATC	215	32	183	14.9	Low	
**PF00023**	IPR002110	ANK	176	113	63	64.2	Low	1
**Domain-coded CDS**			24247	17459	6788	72.0		

a : The domain information was taken from TIGR OSA1 (http://www.tigr.org/tdb/e2k1/osa1/index.shtml).

b : The ratio was calculated as the number of CDS-AE loci/total number of loci.

c : Significances of difference were examined between the CDS-AE proportion for genes encoding the respective domain and that for genes encoding the entire domains examined by the chi-squared test. High (Low) represents the cloning efficiency of genes encoding the respective domain was higher (lower) than the collection efficiency of genes encoding the entire domains examined.

d : Pfam domain data for *E. coli* K12 published at http://www.sanger.ac.uk/Software/Pfam/. A value of 1 indicates that genes encoding the corresponding domain are found in the *E. coli* K12 genome.

It was especially noticed that the CDS-AE proportions were extremely low for three gene families with rice-specific domains (PF03578, PF06654, and PF07197). However, the median signal intensity for CDS-ANE containing PF03578 was approximately 900 (data not shown), suggesting that the inefficient cloning of FL-cDNA from the genes with rice-specific domains might not be due to the lack of active transcription.

It was reported that manipulation and amplification of FL-cDNA clones from some plant virus genomes in *E. coli* were rather difficult because of the instability of viral gene sequences [36]–[38]. It was suggested that the instability of viral genes in *E. coli* resulted from the toxicity of virus-specific gene products [36]–[38]. From these observations we hypothesized that one cause of inefficient FL-cDNA recovery from some gene families could be toxicity of genes encoding protein domains heterologous to *E. coli*. To test this hypothesis, we examined whether the protein domains listed in Table 9 are heterologous to *E. coli*. The protein domains encoded in genes with lower CDS-AE proportions were predominantly heterologous to *E. coli*, while a majority of the domains encoded in genes with higher CDS-AE proportions are also found encoded in genes of *E. coli* (Table 9, Table S5a). Such inefficient production of FL-cDNA from genes encoding domains heterologous to *E. coli* was also observed in Arabidopsis by our analysis. Based on the information available at TAIR6 (http://www.arabidopsis.org/) [25] and SIGnAL database (http://signal.salk.edu/), TAIR6 loci were classified into CDS-AE or CDS-ANE. CDS-AE proportions varied depending on the domains encoded in genes. Gene families encoding domains that are not found in *E. coli* had significantly lower CDS-AE proportions (Table S5b). Therefore, these results support the idea that FL-cDNAs encoding rice-specific or eukaryotic-specific domains might be difficult to clone in *E. coli*. Though we could not analyze the diversity of cloning efficiency between rice and Arabidopsis because the numbers of FL-cDNA clones are drastically different between rice and Arabidopsis (580K and 27K, respectively (http://signal.salk.edu/)), we could not find the diversity of cloning efficiency by organisms (Table S5a and b).

During amplification of foreign genes cloned in *E. coli*, spontaneous deletions and rearrangements of genes which are supposed to be unfavorable to *E. coli* were observed [36], [38]. If a similar event occurs during cloning of rice FL-cDNA, our libraries should contain reassembled FL-cDNA clones. To find out whether such events might have occurred for rice FL-cDNA, we compared the sequences of about 200 short FL-cDNA (<600 bp) with those of the corresponding FL-AE and CDS-AE. The result indicated that some of the short FL-cDNA could be clones which might have been reassembled in *E. coli* (Table 10, Figure S2). It appeared that part of internal exons encoding a protein domain might have been deleted in each FL-cDNA, and most of the domains encoded in the deleted sequences were either absent in *E. coli* or were domains functioning as a gene regulator such as Hsp90 [39]. These results suggested that the cloning of some full-length cDNAs was adversely affected in *E. coli*, and that sequences encoding heterologous domains and those functioning as gene regulators in *E. coli* are the most likely to be affected.

**Table 10:** The list of FL-cDNA in excluded internal sequences

DDBJ accession	Clone length	Chr (a)	Locus length (bp)	TIGR4 locus	Deleted domain (b)	Short name	E.coli (c)
AK058767	492	1	9411	LOC_Os01g05760			
AK058349	527	3	1193	LOC_Os03g08500	IPR001471	AP2-EBPRF	
AK063600	537	3	1190	LOC_Os03g29250	IPR004331	SPX_N	
AK063698	520	4	2725	LOC_Os04g01740	IPR001404	Hsp90	1
AK063751	530	4	2712	LOC_Os04g01740	IPR001404	Hsp90	1
AK060229	524	4	2423	LOC_Os04g21320	IPR006702	DUF588	
AK059443	478	4	1500	LOC_Os04g42020	IPR000315	B-box	
AK105205	592	4	1256	LOC_Os04g58760	IPR006702	DUF588	
AK062513	499	5	1345	LOC_Os05g33220	IPR008390	AWPM-19	
AK062487	526	6	1478	LOC_Os06g10350	IRP001005	Myb_DNA_bd	
AK241732	237	6	4343	LOC_Os06g10600	IPR002913	START_lipid_bd	
AK105179	543	7	1107	LOC_Os07g42610	IPR001841	Zinc finger, RING-type	
AK058489	587	8	1288	LOC_Os08g37370	IPR001993	Mitoch_carrier	
AK063134	432	8	5613	LOC_Os08g45030	IPR003439	ABC_transp_like	1
AK059470	562	9	1928	LOC_Os09g24690	IPR008195	Ribosomal_L34E	
AK063094	444	10	1075	LOC_Os10g39450	IPR001841	Zinc finger, RING-type	
AK070901	457	12	1971	LOC_Os12g29400	IPR004182	GRAM	

a : Chromosome number to which FL-cDNA were mapped in TIGR4.

b : Domains encoded in the sequences excluded from FL-cDNA sequences. The information was obtained from TIGR OSA1.

c : Pfam domain data for *E. coli* K12 published at http://www.sanger.ac.uk/Software/Pfam/. A value of 1 indicates that genes encoding the corresponding domain are found in the *E. coli* K12 genome.

As the results above suggested, rearranged cDNA sequences might have been included in those used to analyze gene structures (e.g., alternative forms, exon-intron analysis). This may have led artificial mismatches in exon-intron structure between the predicted CDS and the FL-cDNA sequences, resulting in misinterpretations of the alternative forms of the loci. Since the redundancy rate for the entire FL-cDNA was only 1.43 (32 775 FL-cDNA/22 943 FL-cDNA loci), it is particularly difficult to confirm the exon-intron structure for most loci by multiple FL-cDNA sequences. Thus, the effect of the *E. coli* bias on recovery and rearrangement of FL-cDNA should be considered in order to avoid any *in silico* artifacts of FL-cDNA.

Our results revealed that the rice FL-cDNA that we collected originated mostly from genes with high transcription activity. In addition, we found significant differences in the recovery of FL-cDNA among gene families presumably due to the bias related to gene function and gene structure in *E. coli* that results in unequal rice FL-cDNA collections. Because rice FL-cDNA clones are produced under cloning biases as described above, it is important to overcome them for accurate and efficient cloning.

The FL-cDNA library contains both protein-coding and non-protein-coding transcripts. Non-coding transcripts have been reported to function as gene regulators [40]; thus, the information on non-coding transcripts is also important to reveal transcription regulation. It has been reported that the number of mRNA-like ncRNA loci was similar to that of protein-coding mRNA loci in mouse [26], and it seems that the number of ncRNA in rice is not many number (FL-AE:FL-NAE = 23 117 : 5 447, Table 2). However, the number of mouse FL-cDNA clones is more than 2000K and is more than three times the number of FL-cDNA clones in rice. So, it does not indicate that the number of ncRNA of rice is less than that of mouse.

Approximately 2400 FL-cDNA clones which were sequenced completely in our library were not able to be mapped on TIGR4. It has previously been reported that some FL-cDNA were mapped in telomere or centromere regions, which have not been assembled in the Pseudomolecules database [29]–[32], [41]. However, the size of un-assembled regions predicted in IRGSP4 is about 4% of the entire genome (http://rgp.dna.affrc.go.jp/E/IRGSP/index.html). This value was lower than the value for unmapped clones (7%) (Table 1). If unmapped clones were mapped on Gap region in genome, we expected the annotation profile of unmapped clones would be similar to that of mapped clones. Therefore, we analyzed unmapped clones for their homology to Arabidopsis CDS using BlastX and classified them according to their similarity (see Gene annotation). Although about 230 of 2400 unmapped clones were confirmed to map on the rice genome by other genome assemblies (Table 1), the proportion of highly-homologous unmapped clones was lower than that of mapped clones and the proportion of no-homologous unmapped clones was higher than that of mapped clones (Table S6). So, we think that about 1000 unmapped clones could be contaminants originating from organisms other than rice.

About 530K out of 578K FL-cDNA clones were derived from about 28.5K loci. Some loci which were not supported by FL-cDNA clones (CDS-ANE) were found to be active in transcription based on the microarray analysis in this study. The signal detection frequency for CDS-ANE loci varied between 26 and 84% depending on the threshold chosen for analysis (Table 5, S3). Thus, we estimate the number of rice genes based on TIGR4 predictions to be between 31.8K (23 193 CDS-AE+[32 697 CDS-ANE×0.265]) and 50.8K (23 193 CDS-AE+[32 697 CDS-ANE×0.843]), excluding non-protein-coding genes such as FL-NAE.

## Materials and Methods

### FL-cDNA library construction, selection of clones, and one-pass sequence analysis

Starting materials used to construct FL-cDNA libraries are indicated in Table S7. Tissues were collected from *Oryza sativa* ssp. *japonica* cv. Nipponbare. Construction of the FL-cDNA libraries, selection of the cDNA clones, and one-pass sequence analysis were described previously [5]. The 5′-, the 3′- or both ends of 578 473 FL-cDNA clones derived from more than 90 libraries were sequenced, and 241 854 5′ end one-pass sequences (FL-EST) and 536 885 3′ end FL-EST were obtained. In addition, 35 187 FL-cDNA clones were completely sequenced. All cDNA sequences were deposited in DNA Data bank of Japan (DDBJ, http://www.ddbj.nig.ac.jp/) (Table S8) [42].

### Mapping of FL-cDNA sequences and FL-EST for rice genomic DNA

FL-cDNA and FL-EST sequences were mapped onto several rice genome assemblies (TIGR4, IRGSP3 and IRGSP4, the Nipponbare sequence by Syngenta, and the 93-11 sequence by BGI) using the BLAT software [42]. The criterion for mapping was >95% identity and >90% coverage. The genomic sequence hit with the highest score was defined as the mapping position. FL-cDNAs and FL-ESTs that mapped to more than one genome sequence with a similarly high score were excluded from analysis.

**Figure 2:**
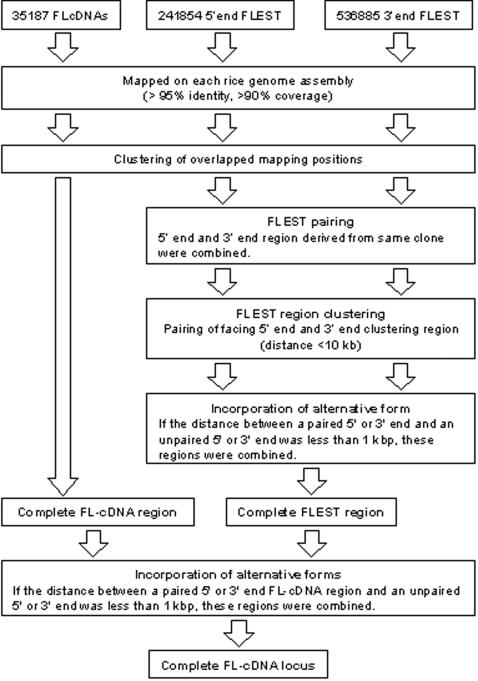
Strategy for mapping of FL-cDNA clones and FL-ESTs, and for definition of FL-cDNA loci.

After mapping the FL-cDNAs and FL-ESTs, clustering analysis was performed to define the FL-cDNA mapped locus. The FL-cDNA and FL-EST mapping results were clustered independently. The 5′ end FL-EST and 3′ end FL-EST mapping results were also clustered independently, and these clustered regions were then combined (Figure 2). These grouping regions were defined as FL-EST regions. The FL-cDNA regions and FL-EST regions were compared to identify the FL-cDNA locus (Figure 2).

### FL-cDNA analysis

The length of the longest ORF was defined as the distance between the nucleotide position (number) of the A in ATG and the last nucleotide before the termination codon. The BlastX algorithm was used for homology search with Arabidopsis genes (TAIR6 (http://www.arabidopsis.org/) [25]).

### Microarray experiment

#### 1. Design of probes and oligo-arrays

FL-cDNA clone sequences (FL-cDNA and FL-EST) were mapped in TIGR Pseudomolecules Release 2 using the BLAT program [43]. FL-cDNA loci were identified with the same criteria as above (i.e., 95% identity and 90% coverage). After mapping, we selected nucleotide sequences from the FL-cDNA locus and CDS of TIGR Pseudomolecules Release 2 to design probes. About 82K 60-mer probe candidates were designed by Agilent Technologies (http://www.home.agilent.com/) considering factors such as the guanine-cytosine (GC) content and melting temperature of the oligonucleotides. The probes were re-mapped in TIGR4 using BLAT [43] according to the same criteria used previously (>95% identity, >90% coverage, non-redundant highest score). We found that 63 455 probes met this criteria and they were mapped onto FL-loci or TIGR loci.

#### 2. Sample preparation

For the microarray experiment, we used total RNA prepared from shoots, roots, panicles, and callus. RNA samples from 10 individual plants were pooled for microarray analysis. Shoots were collected from 14-day-old seedlings of varieties Nipponbare and IR64 grown in pot culture in a greenhouse. Roots were derived from 10-day-old seedlings grown in hydroculture in an incubator. Panicles were collected during the late-boot stage. Callus that originated from the scutellum was incubated for 4 weeks on MS medium [44] that contained 2 mg/L of 2,4-dichlorophenoxyacetic acid and 0.1% Kao and Michayluk Vitamin Solution (Sigma-Aldrich, http://www.sigmaaldrich.com/). These tissues were frozen with liquid N_2_ and stored at −80 °C. Total RNA from the shoots was extracted with Trizol (Invitrogen, https://www.invitrogen.com/) according to the manufacturer's instructions, and was extracted from other tissues using the RNeasy Midi Kit (Qiagen, http://www1.qiagen.com/). The concentration and quality of RNA were checked using the NanoDrop (ND-1000, NanoDrop Technologies, http://www.nanodrop.com/) and the BioAnalyzer (G2938A, Agilent Technologies, http://www.home.agilent.com/), respectively.

#### 3. Labeling, hybridization, and washing

Labeled cRNA was synthesized from 500 ng of total RNA using a Low RNA Input Linear Amplification kit (Agilent Technologies, http://www.home.agilent.com/), according to the manufacturer's protocols. For two-color hybridization, 1 μg of Cyanine 3 (Cy3)-labeled cRNA and 1 μg of Cyanine 5 (Cy5)-labeled cRNA were both used, and the hybridization solution was prepared with an *In Situ* Hybridization Kit Plus (Agilent Technologies, http://www.home.agilent.com/). Hybridization and washing were performed according to the manufacturer's protocols.

#### 4. Scanning and data analysis

The slide images were scanned with the DNA microarray scanner (G2505B, Agilent Technologies, http://www.home.agilent.com/) and the resulting image files were processed to obtain normalized Cy3 and Cy5 signal intensity for each probe using the manufacturer's Feature Extraction version 8.1 software. In this software, normalization of Cy3 and Cy5 signal intensity in each image was performed using locally weighted scatterplot smoothing and rank-consistency methods. In addition, the software also validated whether the signal intensity of each probe was significantly higher than the background signal intensity. The validation was performed in two steps. First, a two-tailed *t*-test was calculated based on the probe's signal and the local background signal and their corresponding errors. If the result was significant at *p*<0.01, the signal was considered to be significantly different from the background. Next, if the raw signal intensity was significantly higher than the local background intensity, the signal intensity was recalculated with the background subtracted. If the resulting signal intensity was greater than 2.6 times the background signal's standard deviation, the signal was also defined as significantly higher than the background signal intensity.

Each sample was hybridized at least four times, but the normalized signal intensity differed among experiments. The differences were attributed to the variation among slides. To reduce the variation, the median of the normalized signal intensity in each array data was adjusted to 1000. After fixation of median intensity, any normalized Cy3 and Cy5 signal intensities >100 000 were considered as the intensity of 100,000, and the dynamic range of signal intensity was defined empirically to be between 0 to 100 000 based on previous experiments. We then calculated the median of the normalized signal intensity for each probe in each sample, and used the resulting median signal intensity to represent the signal intensity of each probe. If only one probe was designed from a FL-cDNA locus or a CDS, the probe's signal intensity was used to define the signal intensity of the locus or CDS. However, if several probes were designed from a FL-cDNA locus or a CDS, the highest signal intensity among the probes was defined as the signal intensity for the locus or CDS. All microarray data are available at the Gene Expression Omnibus (GEO) [45] repository at the National Center for Biotechnology Information (GSE7366 and GSE7374, http://ncbi.nlm.nih.gov/geo/).

## Supporting Information

Figure S1 The results of gene structure analysis. (a) The length distribution of FL-cDNA for FL-AE and FL-NAE. (b) The distribution of open reading frame (ORF) proportions for FL-AE and FL-NAE. (c) The distribution of FL-cDNA locus lengths for FL-AE and FL-NAE. (d) The distribution of locus lengths for CDS-AE and CDS-ANE in TIGR4. (e) The distribution of the number of exons for FL-AE and FL-NAE. (f) The distribution of exon and intron lengths for the respective locus types. (g) The distribution of the number of FL-cDNA clones mapped per single FL-AE and FL-NAE loci.
(8.78 MB TIF)

Figure S2 Sequences encoding specific domains which may have been excluded from FL-cDNA clones. The figures were modified from those found at TIGR OSA1. (a) AK058349 in which the sequence encoding the AP2 domain (IPR001471) was excluded; (b) AK062487 in which the sequence encoding the MYB domain (IPR001005) was excluded.
(8.85 MB DOC)

Table S1 The mapping positions of FL-cDNA in five genome assemblies.
(2.66 MB ZIP)

Table S2 Mapping information of FL-cDNA. a: Information for all loci in TIGR4, b: The list of FL-cDNA loci and mapped FL-cDNA
(3.10 MB ZIP)

Table S3 Signal intensity data for the five samples. a): NB: “Nipponbare”, IR: IR64 b): no-signal: number of loci on which signal intensity was not significant higher than backgroud intensity
(0.02 MB XLS)

Table S4 The correlation coefficient between the density of TE-related locus and the density of locus based on signal intensity under each locus type
(0.01 MB XLS)

Table S5 The relationship between the CDS-AE proportion and domains. a: rice based on TIGR OSA1, b: Arabidopsis based on TAIR6
(0.11 MB ZIP)

Table S6 The frequency of Arabidopsis homologous genes between mapped and un-mapped FL-cDNA clones
(0.02 MB XLS)

Table S7 Library information used to construct the FL-cDNA libraries
(0.03 MB XLS)

Table S8 DDBJ accession IDs for FL-cDNA. a: sequenced 5′ end FL-ESTs and 3′ end FL-ESTs, b: completely sequenced FL-cDNA
(3.58 MB ZIP)
