# Supplementary figures and images for: Gene Organization in Rice Revealed by Full-Length cDNA Mapping and Gene Expression Analysis through Microarray
Source: PLoS One. 2007 Nov 28;2(11):e1235. doi: 10.1371/journal.pone.0001235 (PMC2084198; doi:10.1371/journal.pone.0001235)

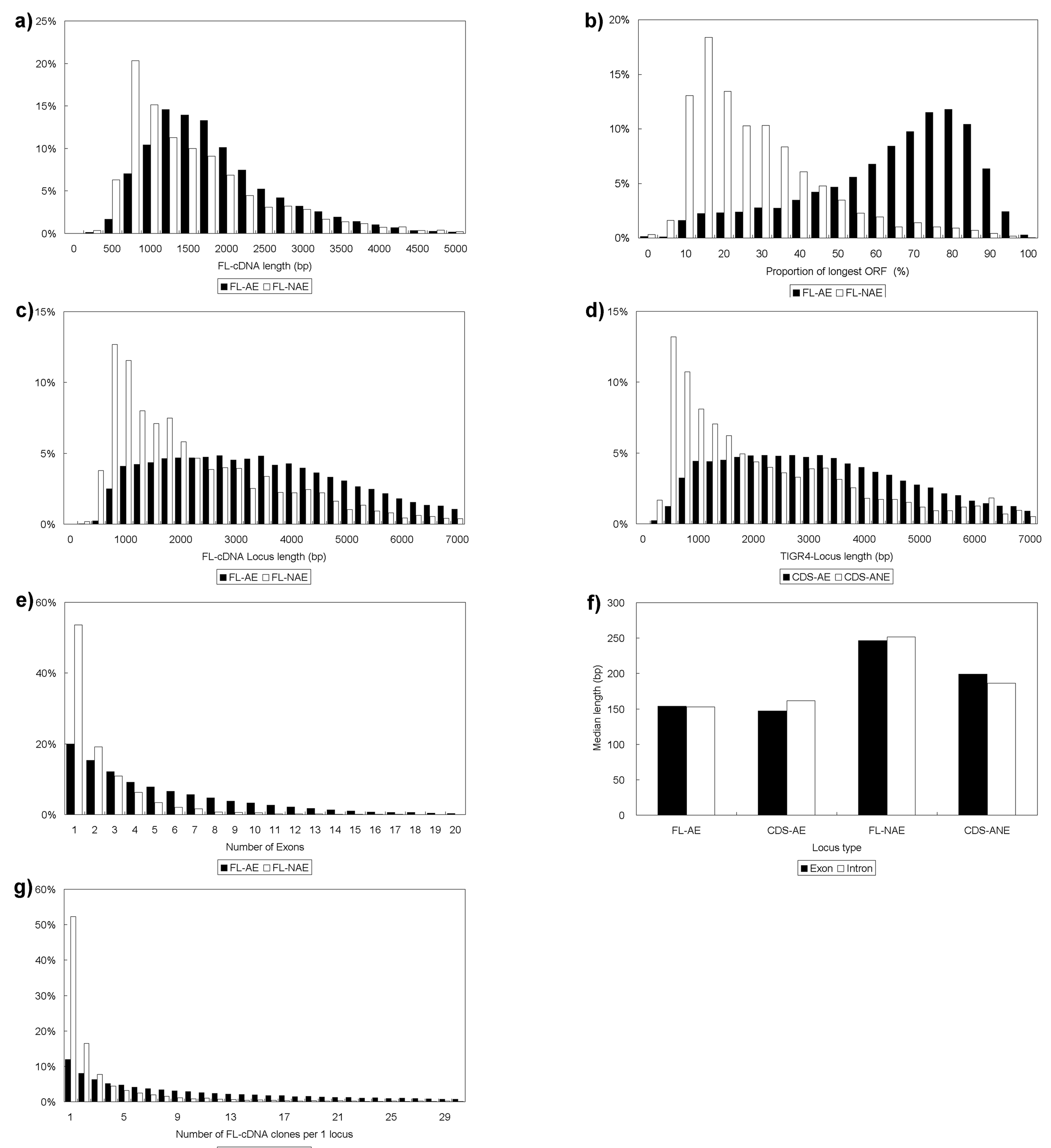

Supplement: Figure S1 — The results of gene structure analysis. (a) The length distribution of FL-cDNA for FL-AE and FL-NAE. (b) The distribution of open reading frame (ORF) proportions for FL-AE and FL-NAE. (c) The distribution of FL-cDNA locus lengths for FL-AE and FL-NAE. (d) The distribution of locus lengths for CDS-AE and CDS-ANE in TIGR4. (e) The distribution of the number of exons for FL-AE and FL-NAE. (f) The distribution of exon and intron lengths for the respective locus types. (g) The distribution of the number of FL-cDNA clones mapped per single FL-AE and FL-NAE loci. (8.78 MB TIF) [file pone.0001235.s001.tif]
