# Supplementary material for: Gene Organization in Rice Revealed by Full-Length cDNA Mapping and Gene Expression Analysis through Microarray
Source: PLoS One. 2007 Nov 28;2(11):e1235. doi: 10.1371/journal.pone.0001235 (PMC2084198; doi:10.1371/journal.pone.0001235)
Supplement: Supplementary file 2 [file pone.0001235.s002.doc]

a)

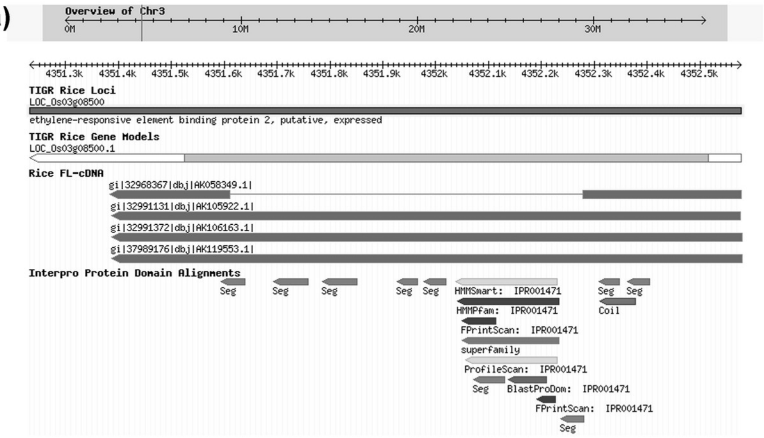
Overview of Chr3
0M
10M
20M
30M
4351.3k 4351.4k 4351.5k 4351.6k 4351.7k 4351.8k 4351.9k 4352k 4352.1k 4352.2k 4352.3k 4352.4k 4352.5k
TIGR Rice Loci
LOC_Os03g08500
ethylene-responsive element binding protein 2, putative, expressed
TIGR Rice Gene Models
LOC_Os03g08500.1
Rice FL-cDNA
gi|32968367|dbj|AK058349.1|
gi|32991131|dbj|AK105922.1|
gi|32991372|dbj|AK106163.1|
gi|37989176|dbj|AK119553.1|
Interpro Protein Domain Alignments
Seg
Seg
Seg
Seg
Seg
HMMSmart: IPR001471
Seg
Seg
HMMPfam: IPR001471
Coil
FPrintScan: IPR001471
superfamily
ProfileScan: IPR001471
Seg
BlastProDom: IPR001471
FPrintScan: IPR001471
Seg

b)

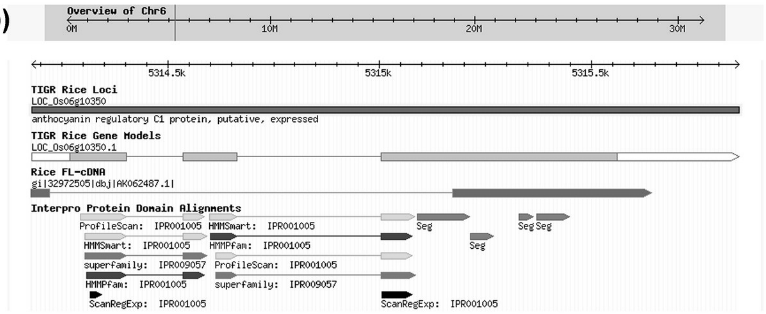
Overview of Chr6
0M
10M
20M
30M
5314.5k
5315k
5315.5k
TIGR Rice Loci
LOC_Os06g10350
anthocyanin regulatory C1 protein, putative, expressed
TIGR Rice Gene Models
LOC_Os06g10350.1
Rice FL-cDNA
gi|32972505|dbj|AK062487.1|
Interpro Protein Domain Alignments
ProfileScan: IPR001005
HMMSmart: IPR001005
Seg
Seg
Seg
HMMSmart: IPR001005
HMMPfam: IPR001005
Seg
superfamily: IPR009057
ProfileScan: IPR001005
HMMPfam: IPR001005
superfamily: IPR009057
ScanRegExp: IPR001005
ScanRegExp: IPR001005
